# Med1 inhibits ferroptosis and alleviates liver injury in acute liver failure via Nrf2 activation

**DOI:** 10.1186/s13578-024-01234-4

**Published:** 2024-04-27

**Authors:** Zi-Ying Lei, Zhi-Hui Li, Deng-Na Lin, Jing Cao, Jun-Feng Chen, Shi-Bo Meng, Jia-Lei Wang, Jing Liu, Jing Zhang, Bing-Liang Lin

**Affiliations:** 1 Department of Infectious Diseases, The Third Affiliated Hospital of Sun Yat-sen University, Guangzhou, 510630 Guangdong China; 2 Guangdong Key Laboratory of Liver Disease Research, The Third Affiliated Hospital of Sun Yat-sen University, Guangzhou, 510630 China; 3 Department of Gastroenterology, The Sixth Affiliated Hospital of Sun Yat-sen University, Guangzhou, 510630 Guangdong China; 4 Key Laboratory of Tropical Disease Control (Sun Yat-sen University), Ministry of Education, Guangzhou, 510080 Guangdong China

**Keywords:** Acute liver failure, Ferroptosis, Mediator complex subunit 1, Nuclear factor erythroid 2-related factor 2, Heme oxygenase-1, NAD(P)H quinone oxidoreductase 1

## Abstract

**Background:**

Extensive hepatocyte mortality and the absence of specific medical therapy significantly contribute to the unfavorable prognosis of acute liver failure (ALF). Ferroptosis is a crucial form of cell death involved in ALF. In this study, we aimed to determine the impact of Mediator complex subunit 1 (Med1) on ferroptosis and its potential hepatoprotective effects in ALF.

**Results:**

Med1 expression is diminished in the liver of lipopolysaccharide (LPS)/D-galactosamine (D-GalN)-induced ALF mice, as well as in hepatocytes damaged by H_2_O_2_ or TNF-α/D-GalN in vitro. Med1 overexpression mitigates liver injury and decreases the mortality rate of ALF mice by ferroptosis inhibition. The mechanism by which Med1 inhibits erastin-induced ferroptosis in hepatocytes involves the upregulation of nuclear factor erythroid 2-related factor 2 (Nrf2) and its downstream antioxidant genes heme oxygenase-1 (HO-1), glutamate cysteine ligase catalytic (GCLC), and NAD(P)H quinone oxidoreductase 1 (NQO1). Furthermore, Med1 overexpression suppresses the transcription of proinflammatory cytokines tumor necrosis factor-α (TNF-α) and interleukin-6 (IL-6) in the liver of mice with LPS/D-GalN-induced ALF.

**Conclusion:**

Overall, our research findings indicate that Med1 suppresses ferroptosis and alleviates liver injury in LPS/D-GalN-induced ALF through the activation of Nrf2. These findings substantiate the therapeutic viability of targeting the Med1-Nrf2 axis as a means of treating individuals afflicted with ALF.

**Graphical Abstract:**

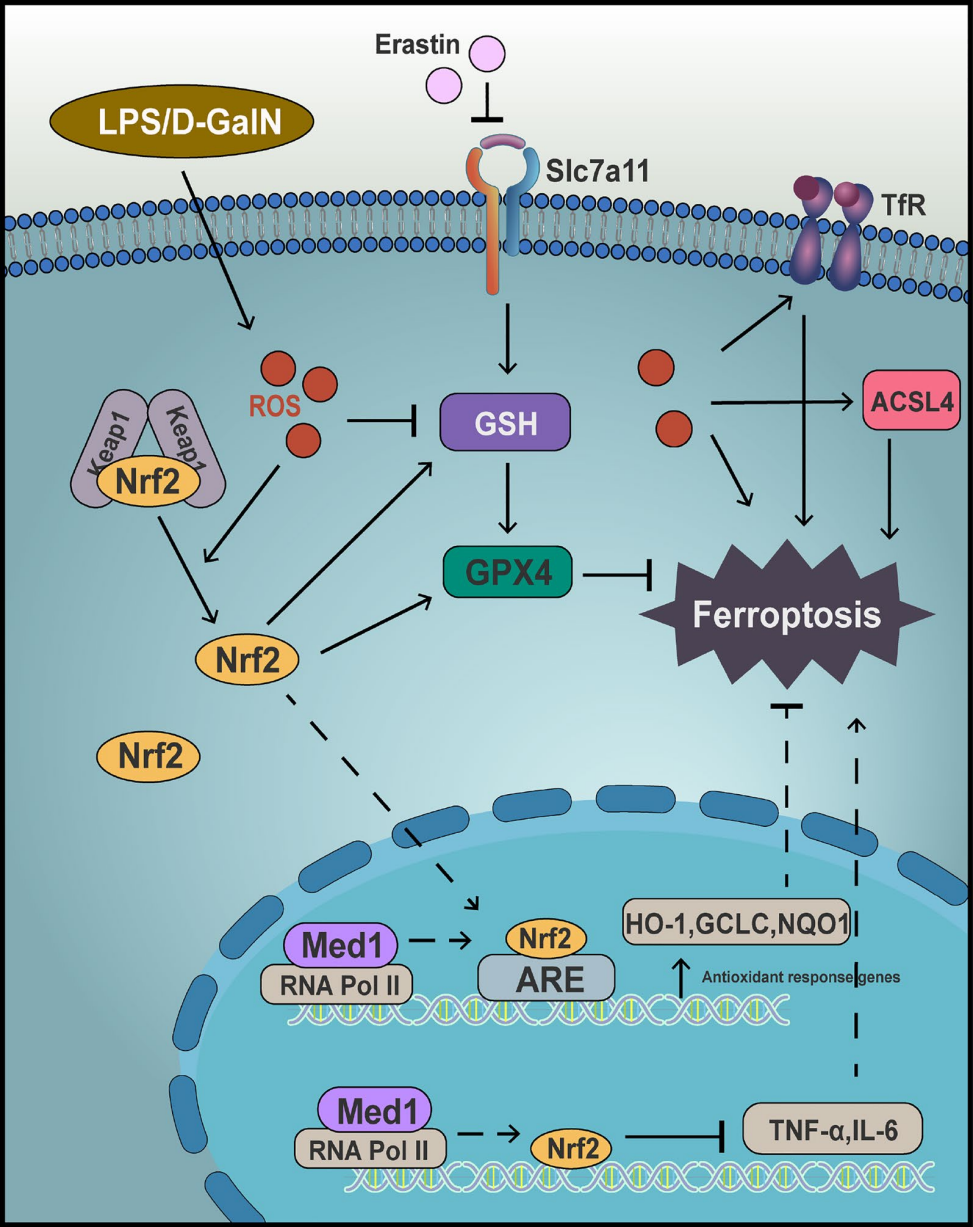

**Supplementary Information:**

The online version contains supplementary material available at 10.1186/s13578-024-01234-4.

## Background

Acute liver failure (ALF) is a severe multifactorial clinical syndrome characterized by extensive hepatocyte necrosis, uncontrolled systemic inflammation, and insufficient hepatic regeneration [1, 2]. Despite significant advancements in medical supportive care, liver transplantation remains the most efficacious intervention for ALF; however, the limited availability of donor livers results in poor survival rates [2]. Hence, it is imperative to develop therapeutic strategies to efficiently attenuate ALF-induced hepatocyte death. In recent years, an increasing body of evidence has emphasized the importance of ferroptosis in the advancement of ALF [3–5].

Ferroptosis, a type of programmed cell death triggered by iron-dependent lipid peroxidation [6], is distinct from apoptosis, necrosis, and autophagy. The process of ferroptosis involves the accumulation of reactive oxygen species (ROS), depletion of glutathione (GSH), and is triggered by the inactivation of glutathione peroxidase 4 (GPX4) [7, 8]. GPX4 is recognized as a critical enzyme that serves to inhibit lipid peroxidation and suppress ferroptosis. Four kinds of markers commonly used to detect ferroptosis include lipid peroxidation, hallmark gene induction, transferrin receptor 1 (TfR1) mobilization, and mitochondria morphological changes. The enzyme Acyl-CoA synthetase long chain family member 4 (ACSL4) plays a key role in incorporating polyunsaturated fatty acids into phospholipids [9], ultimately leading to lipid peroxidation and the generation of malondialdehyde (MDA) and 4-hydroxynonenal (4‐HNE). Additionally, hallmark genes such as prostaglandin-endoperoxide synthase 2 (PTGS2) [8], solute carrier family 7, member 11 (SLC7A11) [10], and ACSL4 are upregulated during the process of ferroptosis. TfR1 facilitates the import of iron into cells, thereby contributing to the iron pool necessary for ferroptosis, and an increase in TfR1 abundance serves as a reliable indicator of ferroptosis [11]. Nrf2 is a prominent mechanism in conferring resistance to ferroptosis, exerting a suppressive effect on this form of cell death by modulating the antioxidant response [7]. Consequently, the targeting of Nrf2 and its downstream genes, such as heme oxygenase-1 (HO-1) and NAD(P)H quinone oxidoreductase 1 (NQO1), presents a promising strategy for combating ferroptosis [12, 13]. Ferroptosis-targeting interventions significantly alleviate ALF symptoms [14, 15]; however, the identification of molecular targets that effectively inhibit ferroptosis and enhance the prognosis of ALF remains elusive.

Mediator complex subunit 1 (Med1), an integral component of the mediator complex, functions as the molecular intermediary connecting gene-specific transcription factors with RNA polymerase II, thereby governing the transcriptional activity of the majority of RNA polymerase II-dependent genes [16, 17]. As a transcriptional co-activator for various nuclear receptors, Med1 plays a crucial role in various biological processes and is indispensable for cellular homeostasis [18]. Our previous research had highlighted Med1's importance in hepatocyte proliferation and liver regeneration [19], prompting further exploration of its therapeutic potential in liver failure. However, rare reports were found on Med1's involvement in liver failure. Some studies have identified Med1’s significant regulatory role in hepatic autophagy and lipid metabolism. The inhibition of Med1 leads to reduced autophagy and mitochondrial function in hepatocytes, along with decreased transcription of genes related to these processes and impaired fatty acid β-oxidation [20]. Med1 is also crucial for mitochondrial function, as its depletion induces mitochondrial biogenesis in C2C12 cells [21]. Furthermore, deficiency of Med1 in macrophages results in heightened levels of ROS [22]. Given that mitochondrial dysfunction, decreased fatty acid oxidation, and ROS accumulation can promote ferroptosis [7, 23, 24], it is hypothesized that Med1 may serve as a protective factor against the initiation of ferroptosis. However, the involvement of Med1 in the regulation of ferroptosis remains unexplored.

Consequently, the objective of this study is to evaluate the impact of Med1 on ferroptosis and its potential hepatoprotective effects in ALF.

## Materials and methods

### Experimental animals and ethics statement

All animal experiments were approved by the Laboratory Animal Ethics Committee of South China Agricultural University (No. 2022d088). C57BL/6 J male mice (5–6 weeks old) were purchased from the Guangdong Sijia Jingda Biotechnology Co., Ltd and randomly assigned to different groups (Additional file 1: Fig. S1). The mice were subjected to a 12-h light/dark cycle and had ad libitum access to food and water for a week before experiments. ALF induction was conducted via intraperitoneal injection of lipopolysaccharide (LPS) (L4391, Sigma-Aldrich) at a dosage of 60 μg/kg and D-galactosamine (D-GalN) (ST1213, Beyotime, China) at a dosage of 800 mg/kg, both dissolved in phosphate-buffered saline (PBS), as previously described [25]. Blood and liver tissue samples were collected at 3 and 6 h post-injection to evaluate the extent of liver damage. In the ferrostatin-1 (Fer-1) group, ferroptosis inhibitor, Fer-1 (10 mg/kg) (HY-15763, MCE), was intraperitoneally administered 1 h before LPS/D-GalN injection.

The full-length mouse Med1 DNA (NM_001080118; 4728 bp) (Additional file 2: Fig. S2) was cloned into the pADM-CMV-C-FH (ADM-FH) vector (Additional file 3: Fig. S3) and packaged into Ad-Med1 adenovirus particles by WZ Biosciences Inc. (Shandong, China). Ad-Med1 (3.6 × 10^9^ PFU) was administered to mice via the tail vein to overexpress Med1 in the liver. Med1 overexpression was confirmed after 3 days using western blotting [19]. Control mice received empty vectors (ADM-FH) adenovirus. ALF was induced in the mice with LPS/D-GalN after successful modeling, and their survival was monitored for 24 h. Five independent experimental replicates were conducted.

### Cell culture and transfection

Normal human liver cell lines, L02 and THLE2, were obtained from Zhong Qiao Xin Zhou Biotechnology Co., Ltd. (Shanghai, China) and authenticated using short tandem repeat analysis. The cells were cultured in Dulbecco’s Modified Eagle Medium (DMEM) (Gibco) supplemented with 10% fetal bovine serum (FBS) (Gibco) and 1% penicillin–streptomycin at 37 °C and 5% CO_2_. THLE2 cells were cultured using the pre-coating collagen culture flasks (356484, Corning) as recommended by ATCC. Primary mouse hepatocytes were isolated and cultured in Roswell Park Memorial Institute (RPMI) 1640 (Gibco) containing 10% FBS as previously described [26, 27]. To establish in vitro models of acute hepatocyte injury, L02 and THLE2 cells were exposed to H_2_O_2_ (1.0 mM) for 2 h, or co-treated with TNF-α (100 ng/ml) (H8916, Sigma-Aldrich) and D-GalN (7.5 mg/ml) for 24 h. Additionally, to induce ferroptosis, the cells were treated with 10 μM erastin (HY15763, MCE) for 24 h.

L02 and THLE2 cells were transfected with Lentivirus overexpressing Med1 (Lv-Med1) or negative control Lentivirus (Lv-NC), that were provided by Genechem (Shanghai, China). To evaluate the impact of Med1 knockdown on ferroptosis, L02 cells and primary mouse hepatocytes were transfected with small interfering RNAs (siRNAs) targeting Med1 (siMed1), or siRNA negative control (siNeg), obtained from GenePharma (Suzhou, China). Forty-eight hours following transfection, the cells were collected. At least three independent experiments were performed.

### Western blot analysis

Protein lysates, obtained from hepatocytes and liver specimens, were electrophoresed on 4–12% precast bis–tris gels (Genscript, Nanjing, China) and subsequently transferred onto polyvinylidene fluoride (PVDF) membranes. The membranes were then blocked using protein-free rapid blocking buffer (EpiZyme, Shanghai, China) and incubated with primary antibodies overnight at 4 °C. Antibodies used were as follows: Med1 (ab243893, Abcam), Nrf2 (D1Z9C) (12721S, CST), HO-1 (R24541, Zen BioScience), ACSL4 (DF12141, Affinity Biosciences), TfR1 (ab269513, Abcam), SLC7A11 (ab175186, Abcam), GPX4 (ab125066, Abcam), NQO1(DF6437, Affinity Biosciences) and β-Tubulin (66,240–1-Ig, Proteintech).

### Real-time PCR analysis

Cellular RNA was isolated using the RNA Quick Purification kit (ESscience, Shanghai, China), and liver tissue RNA was extracted using Trizol reagent (Thermo Fisher, MA, USA), according to the manufacturers’ instructions. Reverse transcription and qPCR were consecutively conducted using the Color Reverse Transcription Kit (with gDNA remover) and 2 × SYBR Green Color qPCR Mix (ROX2 Plus) (EZBioscience, Roseville, USA), according to the manufacturers’ instructions. The expression of the target genes was normalized to that of β-actin gene. The primers utilized in this study are listed in Additional file 4: Table S1.

### MDA, and glutathione (GSH)

The MDA and GSH levels were determined using the MDA Assay Kit (DOJINDO, Japan) and the GSH and GSSG Assay Kit (Beyotime, Shanghai, China), according to the manufacturers’ instructions.

### Histopathological analysis and immunohistochemistry

The liver tissues obtained from mice were fixed in 4% paraformaldehyde for a minimum of 24 h, followed by gradual dehydration and paraffin embedding. Histological and liver injury evaluation was conducted using hematoxylin & eosin (H&E) staining. To detect the expression of 4‐HNE in liver tissues, immunohistochemistry analysis was performed using the HNEJ-2 antibody (ab48506, Abcam), as per the manufacturer’s instructions.

### Statistical analysis

The student’s *t*-test was used to compare groups. Statistical significance set as *p* < 0.05. Analyses were performed using SPSS 22.0 and GraphPad Prism 9.0. The data are presented as Mean ± SD.

## Results

### Med1 is downregulated in ALF hepatocytes

**Fig. 1:**
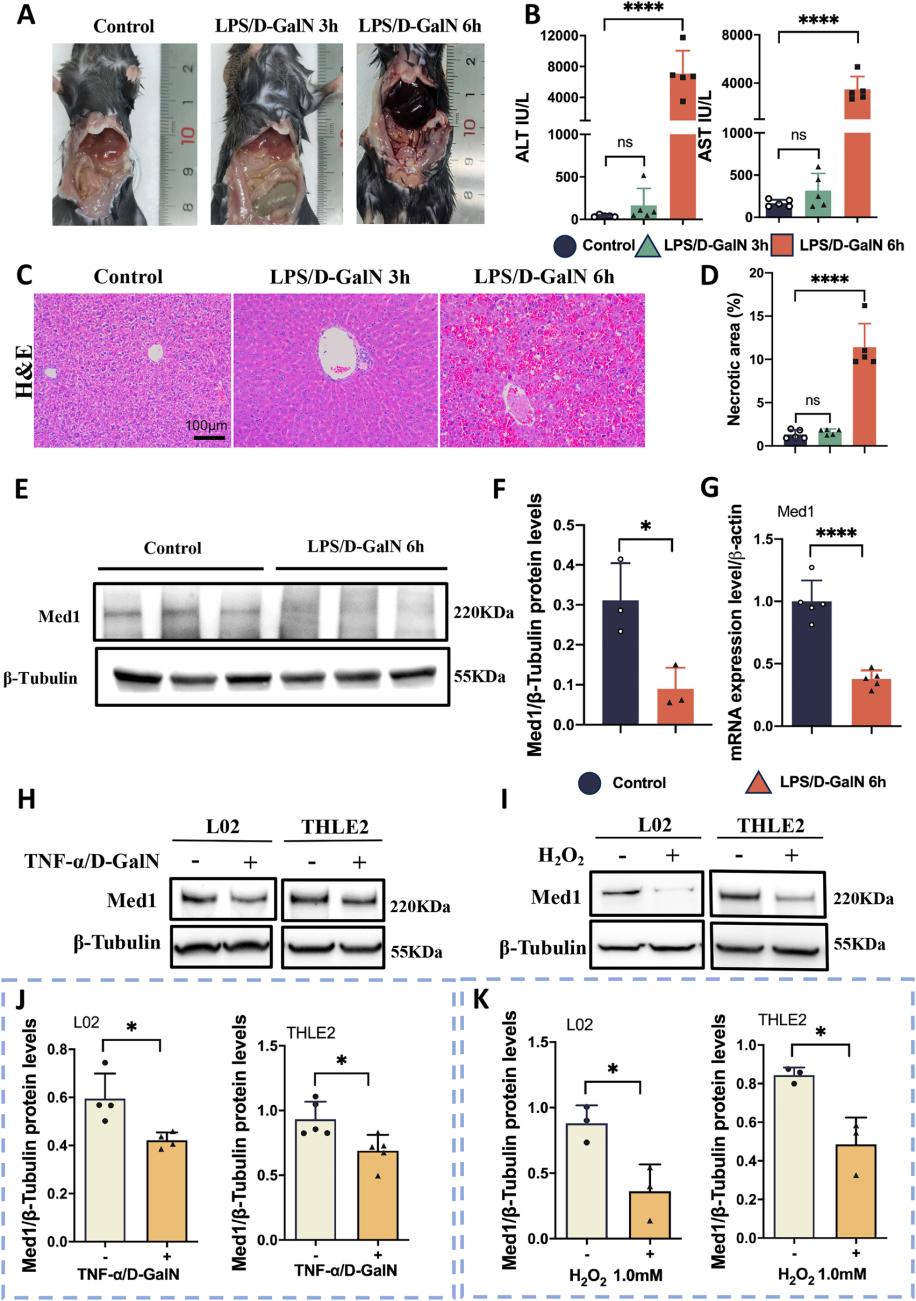
Med1 expression is downregulated in ALF hepatocytes. **A** Representative pictures of mice treated with PBS or LPS (60 μg/kg)/D-GalN (800 mg/kg) for 3 and 6 h (n = 5). **B** Serum levels of ALT and AST were significantly elevated in ALF mice (n = 5). **C** H&E staining of liver tissues from mice treated with PBS or LPS/D-GalN for 3 and 6 h (n = 5). Scale bar: 100 μm. **D** Quantification of necrotic area between three groups (n = 5). **E** Protein levels of Med1 in liver tissues from mice treated with PBS or LPS/D-GalN for 6 h (normalized to β-Tubulin) measured by Western blot and **F** relative grayscale analysis using Image J software (n = 3). **G** mRNA levels of Med1 in liver tissues from mice treated with PBS or LPS/D-GalN for 6 h (normalized to β-actin), assessed by RT-qPCR (n = 5). **H**–**K** Protein levels of Med1 in L02 and THLE2 cells following stimulation with TNF-α (100 ng/ml)/D-GalN (7.5 mg/ml) for 24 h or H_2_O_2_ (1.0 mM) for 2 h evaluated using Western blot and quantified using Image J software (n = 3–4). Data are presented as mean ± SEM. Statistical analysis was performed using Student’s *t*-test, *p < 0.05, ***p < 0.001, ****p < 0.0001

The LPS/D-GalN-induced ALF model resulted in mortality begin at 5–6 h, consistent with our previous findings [25]. Notably, compared with livers from the normal control group, no significant differences were observed in the appearance of the liver harvested from the ALF group at 3 h. However, at 6 h, the harvested liver from the ALF group exhibited notable swelling and congestion (Fig. 1A). Additionally, at 6 h, alanine aminotransferase (ALT) and aspartate aminotransferase (AST) were significantly elevated (Fig. 1B). In addition, H&E staining revealed mild edema of hepatocytes at 3 h, whereas at 6 h, extensive liver damage (characterized by widespread hemorrhage and necrosis) was observed (Fig. 1C, D). These findings indicate the successful establishment of the ALF mouse model 6 h post-LPS/D-GalN exposure. To determine the role of Med1 in ALF, we evaluated Med1 expression in the livers of ALF mice. Our analysis revealed a decrease in both protein and mRNA levels of Med1 in the livers of ALF mice (Fig. 1E–G). Furthermore, we assessed Med1 expression in damaged hepatocytes in vitro. Remarkably, the protein levels of Med1 were diminished in hepatocytes following TNF-α/D-GalN or H_2_O_2_ stimulation (Fig. 1H–K).

### Med1 overexpression alleviated LPS/D-GalN-induced ALF in mice

**Fig. 2:**
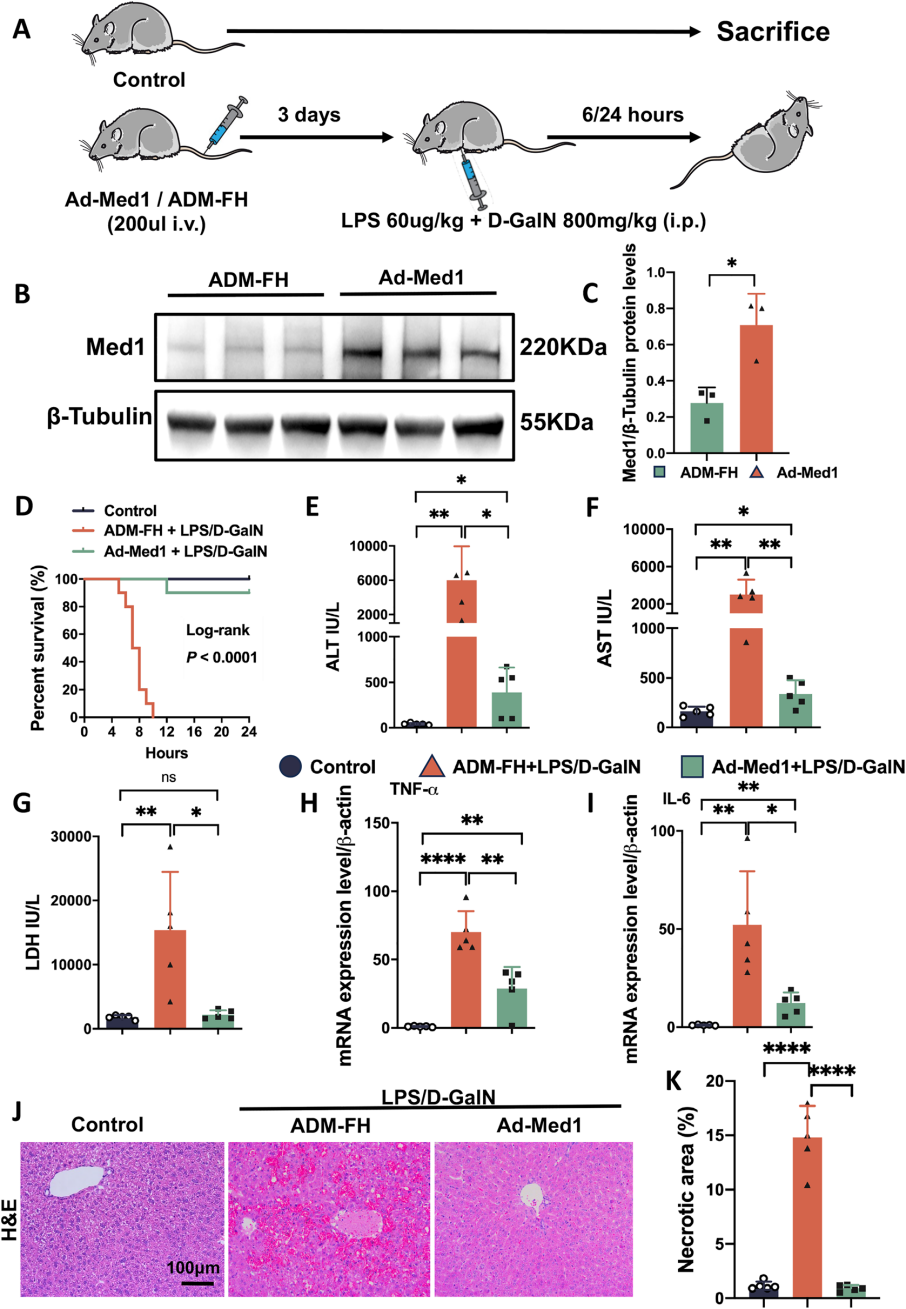
Med1 overexpression alleviated LPS/D-GalN-induced ALF in mice. **A** Wild-type mice injected with Ad-Med1 (3.6 × 10^9^ pfu in 200 μl) or empty vector ADM-FH via the tail vein to establish the Med1 over-expression model or controls, respectively, 3 days later intraperitoneally injected with LPS (60 μg/kg)/D-GalN (800 mg/kg) to induce ALF. **B**, **C** Western blotting and relative grayscale analysis confirmed Med1 overexpression in the liver of Ad-Med1 mice (n = 3). **D** Med1 overexpression significantly improved the 24-h survival rate of ALF (n = 10). **E**–**G** Serum levels of ALT, AST, and LDH were elevated after LPS/D-GalN injection in the ADM-FH group, but significantly decreased in the Ad-Med1 group (n = 5). **H**, **I** mRNA levels of TNF-α and IL-6 in liver tissues were elevated after LPS/D-GalN injection in the ADM-FH group, but significantly decreased in the Ad-Med1 group (n = 5). **J**–**K** H&E staining of liver tissues and quantitation of necrosis area between groups (n = 5). Scale bar: 100 μm. Data are presented as mean ± SEM. Statistical analysis was performed using the Student’s *t*-test, *p < 0.05, **p < 0.01, ****p < 0.0001

Since Med1 was downregulated in the liver of LPS/D-GalN-induced ALF mice, we aimed to investigate the potential protective effects of Med1 overexpression on liver injury. To establish the Med1 overexpression mouse model, Ad-Med1 particles were administered via the tail vein (Fig. 2A). Med1 overexpression in the liver of Ad-Med1 mice was confirmed through Western blot analysis (Fig. 2B, C). Subsequently, ALF was induced using LPS/D-GalN (Fig. 2A). In the ADM-FH group, mice began to die within 5 h of LPS/D-GalN exposure, with all mice succumbing to ALF within 10 h. In contrast, only one mouse in the Ad-Med1 group died within 24 h, suggesting that Med1 overexpression significantly reduced the 24-h ALF mortality rate (*P* < 0.0001). Additionally, Med1 overexpression led to a decrease in serum ALT, AST, and lactate dehydrogenase (LDH) levels (Fig. 2E–G). Furthermore, Med1 overexpression was associated with TNF-α and IL-6 downregulation in the liver (Fig. 2H, I). In contrast, following LPS/D-GalN exposure, liver sections from the Ad-Med1 group displayed reduced hepatic congestion and hemorrhage (Fig. 2J, K). These findings strongly indicate that Med1 overexpression effectively alleviates the progression of LPS/D-GalN-induced ALF.

### Fer-1 inhibits ferroptosis but does not alleviate inflammation in ALF

**Fig. 3:**
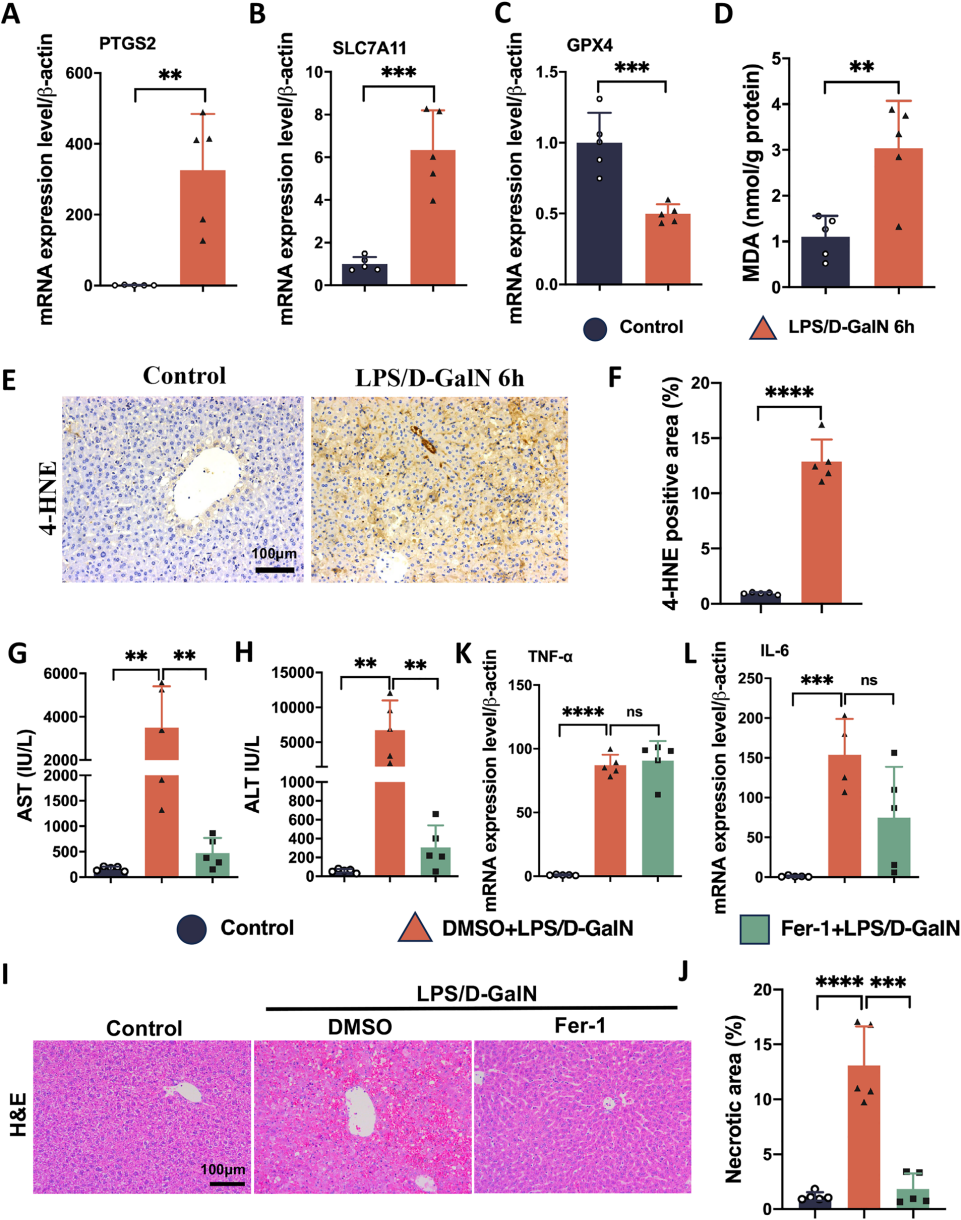
Ferroptosis is an important driver of LPS/D-GalN-induced ALF. **A**, **B** mRNA levels of PTGS2 and SLC7A11 increased in liver tissues of the ALF group (n = 5). **C** GPX4 mRNA expression decreased in liver tissues of the ALF group (n = 5). **D** MDA elevated in liver tissues of the ALF group (n = 5). **E**, **F** 4-HNE immunohistochemistry staining and quantitative results showed that 4-HNE increased in liver tissues of the ALF group (n = 5). **G**, **H** Serum levels of AST and ALT were elevated in ALF mice, but significantly decreased in Fer-1 (10 mg/kg) treatment group (n = 5). **I**, **J** H&E staining of liver tissues and quantitation of necrosis area showed that Fer-1 alleviated liver damage (n = 5). **K**, **L** TNF-α and IL-6 mRNA expression between groups (n = 4–5). Data are presented as mean ± SEM. Statistical analysis was performed using Student’s *t*-test, **p < 0.01, ***p < 0.001, ****p < 0.0001

Extensive research has demonstrated that ferroptosis plays a critical role in the cellular demise observed in liver failure models [4, 28]. To determine the involvement of ferroptosis in LPS/D-GalN-induced ALF in mice, we conducted qPCR analysis to assess the expression of ferroptosis-associated marker genes in the liver of ALF mice. PTGS2 and SLC7A11 gene expression was significantly upregulated (Fig. 3A, B), while GPX4 expression was noticeably downregulated (Fig. 3C). Subsequently, we determined the extent of lipid peroxidation in hepatic tissues. MDA and 4-HNE in the ALF group were significantly higher than that of the control group (Fig. 3D–F). To further explore the potential involvement of ferroptosis in LPS/D-GalN-induced ALF, Fer-1 was administered to determine the effects of ferroptosis inhibitors in ALF. Fer-1 effectively mitigated the elevation of AST and ALT (Fig. 3G, H). In addition, following 6 h of LPS/D-GalN exposure, Fer-1 alleviated liver congestion and hepatocyte necrosis (Fig. 3I, J). These findings suggest that ferroptosis plays a significant role in the development of LPS/D-GalN-induced liver injury in mice. However, it should be noted that Fer-1 administration did not result in TNF-α or IL-6 mRNA downregulation (Fig. 3K, L).

### Overexpression of Med1 alleviated LPS/D-GalN-induced ferroptosis in ALF

**Fig. 4:**
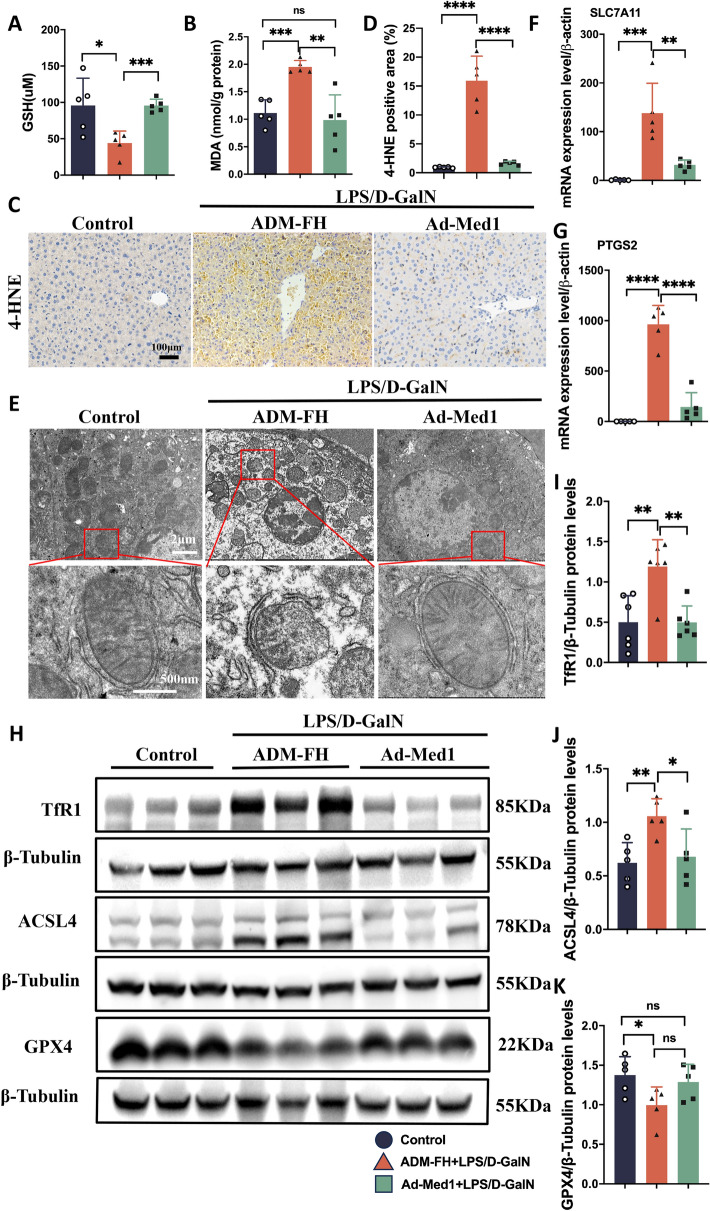
Med1 overexpression alleviated LPS/D-GalN-induced ferroptosis in ALF. **A** Med1 overexpression alleviated the depletion of GSH in the liver of mice following LPS/D-GalN injection (n = 5). **B** Med1 overexpression inhibited the generation of MDA in the liver of mice after LPS/D-GalN injection (n = 5). **C**, **D** Immunohistochemistry staining and quantitative analysis showed that 4-HNE increased in the ADM-FH group, but not in Ad-Med1 group after LPS/D-GalN injection (n = 5). **E** Transmission electron micrographs of mitochondria from ALF mice pretreated with Ad-Med1 or ADM-FH, compared to normal controls, representative images from n = 3 mice per group, Scale bars: up, 2 µm; down, 500 nm. **F**, **G** mRNA levels of SLC7A11 and PTGS2 in liver tissues were elevated after LPS/D-GalN injection in the ADM-FH group, but significantly decreased in the Ad-Med1 group (n = 5). **H** Western blotting and **I**–**K** relative grayscale analysis by Image J software to assess the protein levels of TfR1, ACSL4, and GPX4 in the liver between groups (n = 5). Data are presented as mean ± SEM. Statistical analysis was performed using Student’s *t*-test, *p < 0.05, **p < 0.01, ***p < 0.001, ****p < 0.0001

To elucidate the role of Med1 in ferroptosis, we evaluated the expression of multiple ferroptosis-linked markers in Ad-Med1 and ADM-FH mice co-injected with LPS/D-GalN. Med1 overexpression successfully alleviated GSH depletion in the mice liver (Fig. 4A). Furthermore, the expression of MDA and 4-HNE in the liver of Ad-Med1 mice decreased significantly (Fig. 4B–D). Subsequently, an electron microscopy analysis was conducted to examine the mitochondria in the liver. Compared with the control group, electron microscopy revealed that the mitochondrial structure in liver tissues obtained from the ADM-FH group were altered; mitochondrial swelling, outer mitochondrial membrane rupture, and disorganized cristae were observed. Med1 overexpression improved the aforementioned morphological phenotype (Fig. 4E). Additionally, we found that Med1 overexpression restricted LPS/D-GalN-induced SLC7A11 and PTGS2 upregulation (Fig. 4F, G). Furthermore, hepatic expression of TfR1 and ACSL4 was a notably reduced in the Ad-Med1 group (Fig. 4H–J). In contrast, GPX4 expression was diminished in the ADM-FH group (Fig. 4H, K). These findings suggest that Med1 overexpression effectively mitigated the occurrence of LPS/D-GalN-induced ferroptosis in ALF.

### Med1 inhibits erastin-induced ferroptosis in hepatocytes via Nrf2 activation

**Fig. 5:**
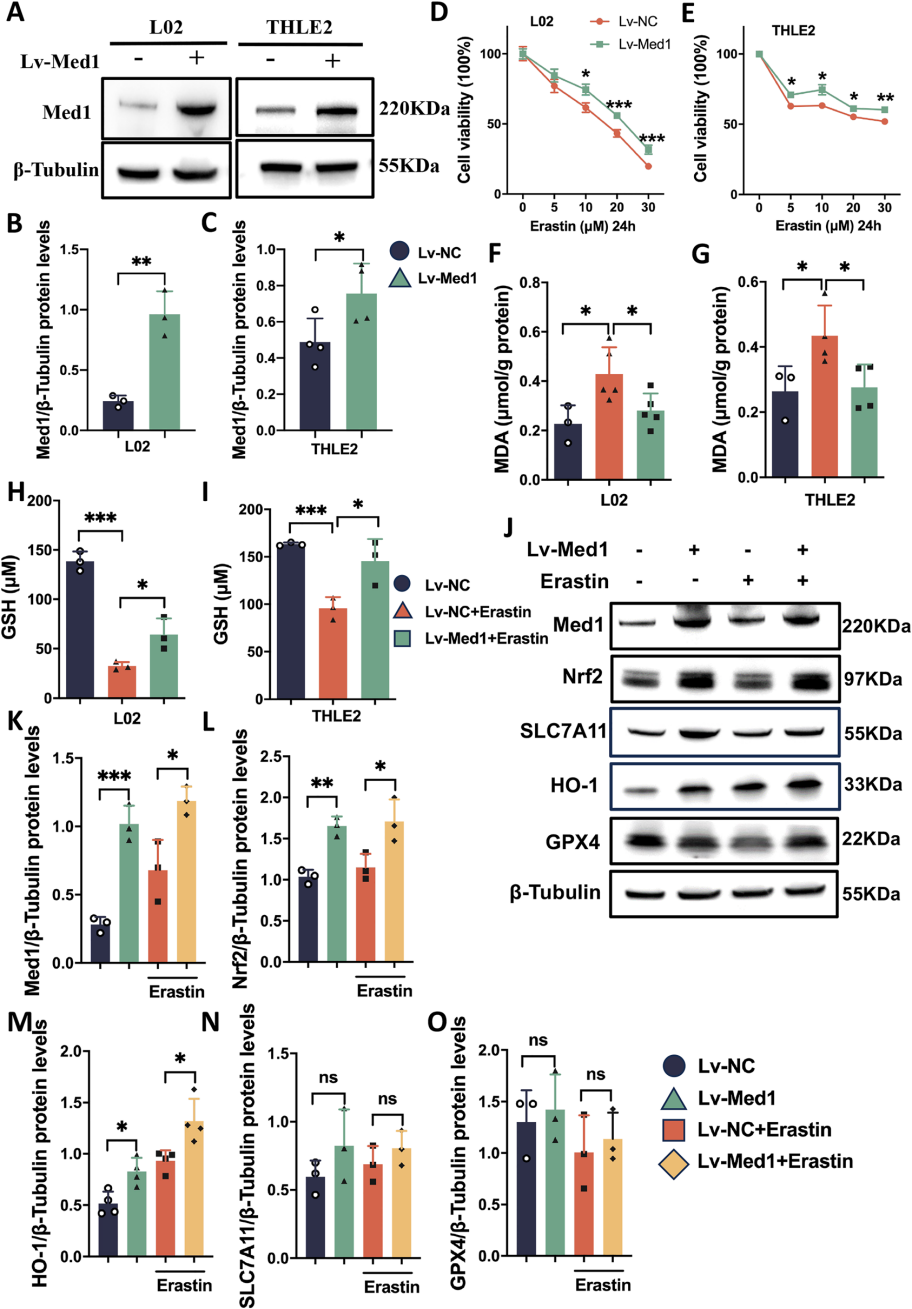
Med1 inhibits erastin-induced ferroptosis in hepatocytes. **A**–**C** Western blotting and relative grayscale analysis by Image J software confirmed Med1 overexpression in L02 and THLE2 cells transfected with Lv-Med1 (n = 3–4). **D**, **E** Relative cell viability of L02 and THLE2 transfected with Lv-Med1 or Lv-NC confirmed using the CCK-8 assay (n = 6–8). **F**–**G** MDA increased following erastin exposure (10 μM, 24 h) in Lv-NC cells, but not in Ad-Med1 cells (n = 3–5). **H**, **I** The level of GSH decreased following erastin exposure in Lv-NC cells, Med1 overexpression inhibited GSH depletion (n = 3). **J** Western blotting and relative grayscale analysis by Image J software **K**–**O** revealed the protein level of Med1, Nrf2, HO-1, SLC7A11, and GPX4 in Lv-Med1 (L02) and Lv-NC (L02) cells treated with or without erastin (n = 3–4). Data are presented as mean ± SEM. Statistical analysis was performed using Student’s *t*-test, *p < 0.05, **p < 0.01, ***p < 0.001

To evaluate the potential protective effects of Med1 against ferroptosis, we established an in vitro hepatocyte ferroptosis model using erastin. Western blot analysis was used to confirm Med1 overexpression in two Lv-Med1-transfected cell lines (L02 and THLE2) (Fig. 5A–C). Upon erastin exposure, cell viability in Lv-Med1 cells was significantly higher than that in Lv-NC cells (Fig. 5D, E). Additionally, Med1 overexpression decreased MDA production (Fig. 5F, G), and increased GSH (Fig. 5H, I) in erastin-treated hepatocytes. These findings support the in vivo findings; Med1 plays an inhibitory role in erastin-induced ferroptosis in hepatocytes.

**Fig. 6:**
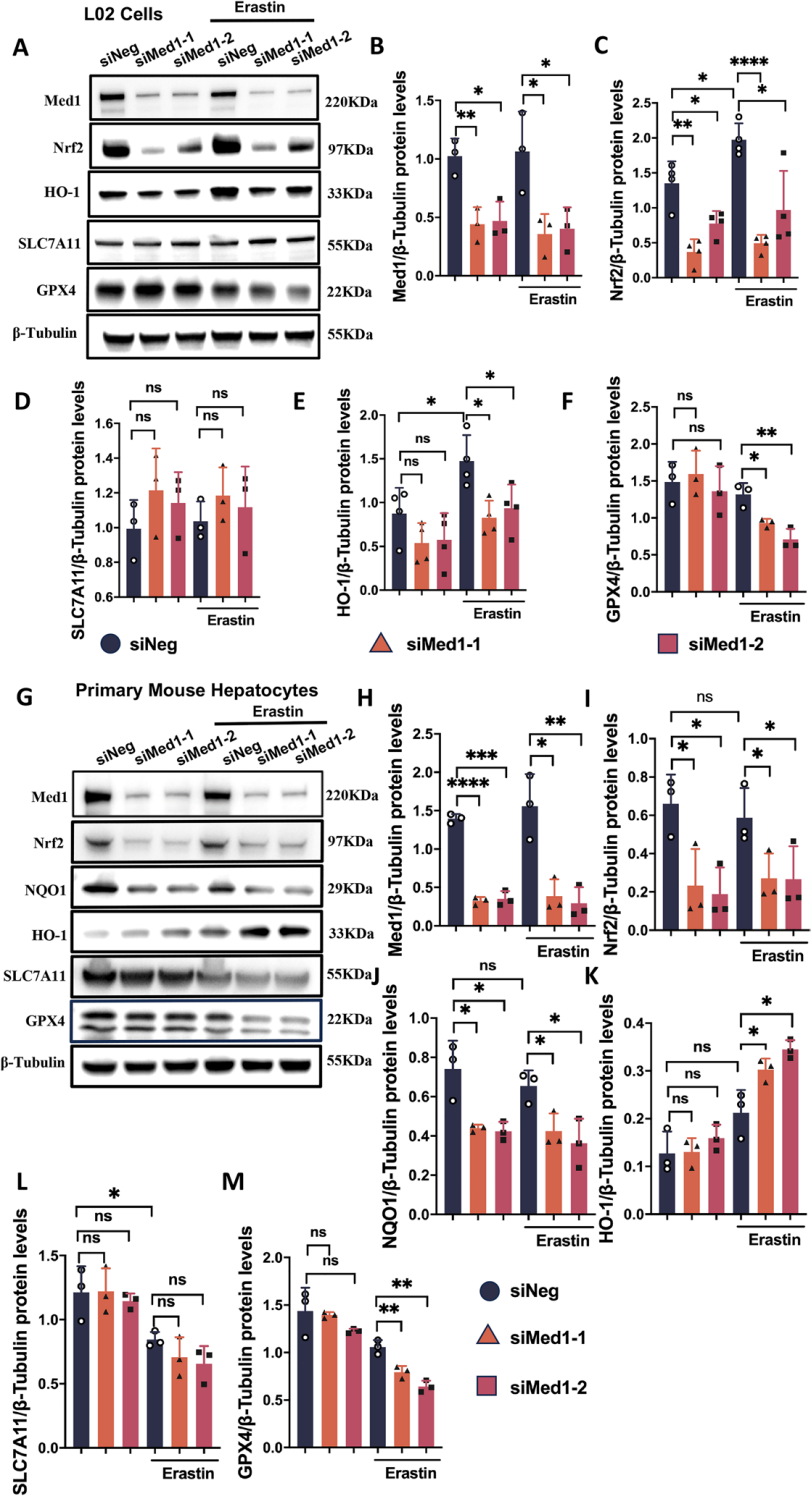
Med1 knockdown affects ferroptosis-related molecules. **A** Western blotting and relative grayscale analysis by Image J software verified siMed1 knockdown in L02 cells (**B**), and assessed Nrf2 (**C**), SLC7A11 (**D**), HO-1 (**E**), and GPX4 (**F**) protein expression in siNeg and siMed1 cells treated with or without erastin (n = 3–4). **G** Western blotting and relative grayscale analysis by Image J software verified siMed1 knockdown in primary mouse hepatocytes (**H**), and assessed Nrf2 (**I**), NQO1 (**J**), HO-1 (**K**), SLC7A11 (**L**), and GPX4 (**M**) protein expression in siNeg and siMed1 cells treated with or without erastin (n = 3). Data are presented as mean ± SEM. Statistical analysis was performed using Student’s *t*-test, *p < 0.05, **p < 0.01, ***p < 0.001, ****p < 0.0001

In order to gain a deeper understanding of the regulatory mechanism of Med1 in ferroptosis, we evaluated the expression of ferroptosis-related molecules. Interestingly, in Lv-Med1 cells, Nrf2 expression increased significantly regardless of the presence or absence of erastin stimulation (Fig. 5J–L). HO-1, a gene primarily regulated by Nrf2, exhibited a similar trend (Fig. 5J, M). However, there were no notable differences in SLC7A11 or GPX4 expression between Lv-Med1 cells and Lv-NC cells (Fig. 5J, N, O). Subsequently, two distinct siRNAs were used to inhibit Med1 expression in L02 cells. Western blot analysis revealed that the expression of both Med1 and Nrf2 in siMed1 cells were significantly reduced (Fig. 6A–C). However, SLC7A11, HO-1, and GPX4 protein expression was comparable between the siMed1 and siNeg groups (Fig. 6A, D–F). Furthermore, erastin administration stimulated Nrf2 and HO-1 expression in siNeg cells, but not in siMed1 cells (Fig. 6A, C, E). Moreover, upon erastin exposure, the expression of GPX4 was significantly diminished in siMed1 cells (Fig. 6A, F). The siMed1 experiment was replicated in primary mouse hepatocytes, revealing that the expression patterns of Nrf2 and GPX4 proteins were consistent with those of L02 cells (Fig. 6G–I, M). While HO-1 exhibited divergent behavior between the two cell lines (Fig. 6G, K), NQO1, another downstream antioxidant gene regulated by Nrf2, showed significant downregulation in siMed1 cells (Fig. 6G, J). Additionally, the expression of SLC7A11 was also unaffected by Med1 but decreased with erastin treatment (Fig. 6G, L). These results indicate that Med1 plays a crucial role in the activation of Nrf2 and its downstream targets.

**Fig. 7:**
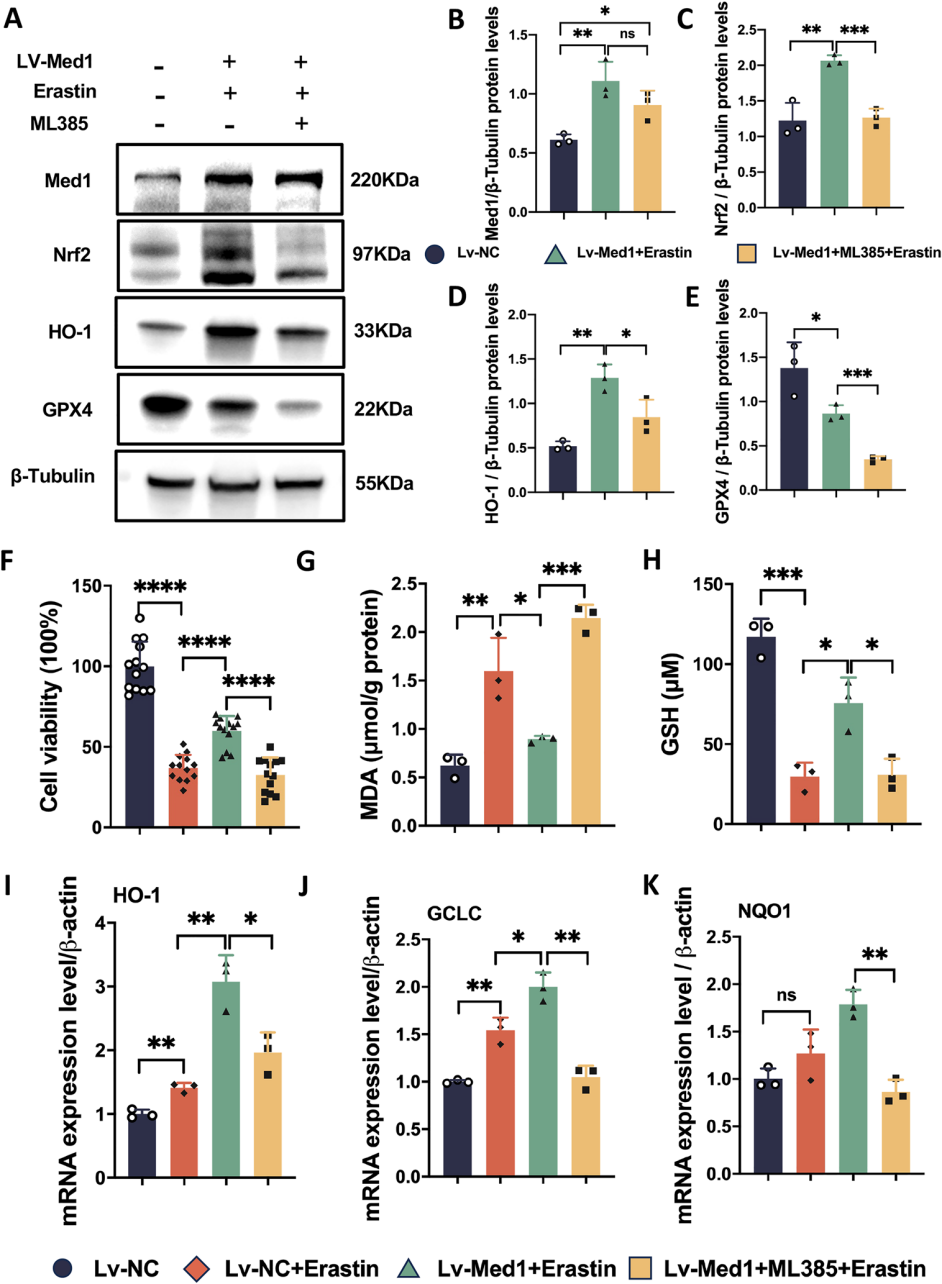
Ferroptosis inhibition by Med1 was reversed by ML385. **A** Western blotting and relative grayscale analysis by Image J software assessed the protein levels of Med1 (**B**), Nrf2 (**C**), HO-1 (**D**), and GPX4 (**E**), Lv-Med1 cells were treated with ML385 (10 μM for 12 h) before the treatment of erastin (10 μM for 24 h) in Lv-Med1 + ML385 + Erastin group (n = 3). **F** Relative Lv-NC and Lv-Med1 cell viability assessed using the CCK-8 assay (n = 13). **G** MDA and **H** GSH levels detected in Lv-NC and Lv-Med1 cells treated with or without eratstin and ML385 (n = 3). **I**–**K** mRNA levels of HO-1, GCLC, and NQO1 increased in Lv-Med1 cells following erastin treatment, but decreased upon the administration of ML385 (n = 3). Data are presented as mean ± SEM. Statistical analysis was performed using Student’s *t*-test, *p < 0.05, **p < 0.01, ***p < 0.001, ****p < 0.0001

To investigate whether the inhibition of ferroptosis by Med1 is dependent on Nrf2, we utilized the Nrf2 inhibitor ML385 to suppress the expression of Nrf2 in Lv-Med1 cells. ML385 treatment inhibited the anticipated Nrf2 and HO-1 upregulation in Lv-Med1 cells (Fig. 7A–D), and GPX4 decreased significantly under these conditions (Fig. 7E). The Cell Counting Kit-8 (CCK-8) assay demonstrated that Med1 overexpression improved cell viability. However, this beneficial effect was counteracted by the presence of ML385 (Fig. 7F). Furthermore, ML385 reversed the protective ferroptosis effects exerted by Med1. This was evident through the observed increase in MDA levels and decrease in GSH levels (Fig. 7G, H). Additionally, the expression of Nrf2 downstream antioxidant genes HO-1, Glutamate cysteine ligase catalytic (GCLC), and NQO1 increased significantly in Lv-Med1 cells following erastin treatment. However, the effects were reversed upon ML385 administration (Fig. 7I–K). These findings suggest that Med1 promotes the expression of Nrf2 and its downstream genes.

**Fig. 8:**
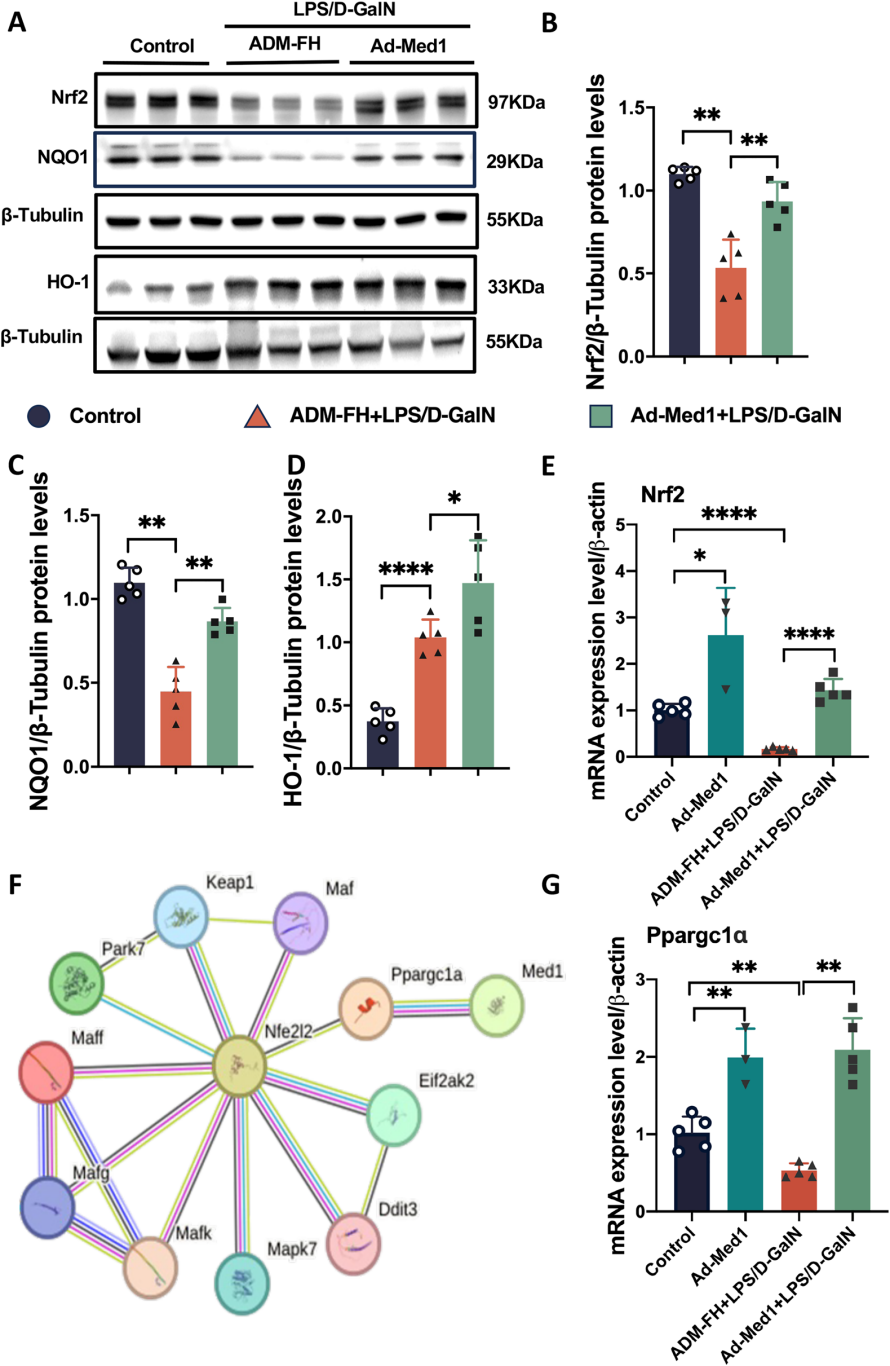
Med1 inhibits ferroptosis via Nrf2 activation. **A** Western blotting and relative grayscale analysis by Image J software assessed the protein levels of Nrf2 (**B**), NQO1 (**C**), and HO-1 (**D**) in Ad-Med1 and ADM-FH groups after LPS/D-GalN injection (n = 5). **E** mRNA level of Nrf2 between groups (n = 3–5). **F** The interaction of top ten predicted functional partners of Nrf2 (Nfe2l2) and Med1 shown in the STRING database (https://string-db.org/). **G** mRNA level of Ppargc1α between groups (n = 3–5). Data are presented as mean ± SEM. Statistical analysis was performed using Student’s *t*-test, *p < 0.05, **p < 0.01, ****p < 0.0001

Consistent with the in vitro findings, upon LPS/D-GalN-exposure, the expression of Nrf2, NQO1, and HO-1 proteins were upregulated in the liver of the Ad-Med1 group (Fig. 8A–D). To explore how Med1 promoted Nrf2 expression, the mRNA levels of Nrf2 were assessed. Nrf2 mRNA levels decreased in the ADM-FH group following LPS/D-GalN treatment, while they increased in the Ad-Med1 group regardless of LPS/D-GalN stimulation (Fig. 8E), suggesting that Med1 overexpression facilitated the transcription of the Nrf2 gene. Nevertheless, the precise mechanism underlying the interaction between Med1 and Nrf2 remains elusive. The STRING database (https://string-db.org/) was utilized to analyze molecular interactions, revealing that among the top ten predicted functional partners of Nrf2 (also known as Nfe2l2) in mice with a confidence level of 0.700, only peroxisome proliferator-activated receptor gamma co-activator 1 alpha (Ppargc1α) exhibited interaction with Med1 (Fig. 8F). Furthermore, the mRNA expression of Ppargc1α was upregulated in Ad-Med1 mice regardless of LPS/D-GalN stimulation (Fig. 8G), suggesting a potential role of Med1 in enhancing Nrf2 transcriptional activity through Ppargc1α.

## Discussion

Extensive hepatocyte mortality and the lack of specific medical therapy are key factors that contribute to the unfavorable prognosis of ALF [1, 2]. Consequently, there is an urgent need to develop effective strategies to mitigate hepatic cell death. The LPS/D-GalN-induced ALF is a widely accepted experimental model that accurately replicates endotoxin-induced acute liver damage observed in humans [28, 29]. Recently, the importance of ferroptosis in ALF has been highlighted [4, 28]. In the liver of mice with ALF induced by acetaminophen or LPS/D-GalN, the expression of classical markers associated with ferroptosis, such as PTGS2, SLC7A11 genes, and lipid peroxidation products (4-HNE and MDA) increase [4, 30]. Furthermore, in sepsis-induced liver failure, ferroptosis-associated genes are upregulated [31], suggesting an important role of ferroptosis in the development of liver failure due to diverse etiologies.

It has been extensively demonstrated that therapeutic interventions targeting ferroptosis can attenuate liver injury [4, 5, 14, 30, 32, 33]. In our study, it was likewise found that ALF-associated liver damage was reversed with Fer-1 (a ferroptosis specific inhibitor) [6] administration. Med1 not only inhibited ferroptosis occurring in vivo by LPS/D-GalN-induced ALF, but also inhibited erastin-induced ferroptosis in hepatocytes in vitro. This suggests that the alleviation of LPS/D-GalN-induced ALF by Med1 is most likely attributable to the inhibition of ferroptosis. Med1 is indispensable in liver regeneration after hepatectomy and stimulates hepatocyte DNA synthesis [19, 34]. Physiologically, Med1 is a well-established coactivator of multiple nuclear receptors, and is involved in critical processes such as cellular differentiation, development, and metabolism [16, 18]. Additionally, Med1 potentially mitigates atherosclerosis by promoting the polarization of macrophage M2 and suppressing the ROS generation [22, 35]. We found that TNF-α and IL-6 was reduced in the liver of Ad-Med1 mice following LPS/D-GalN exposure, suggesting that Med1 has an inhibitory impact on inflammation. However, the mechanisms by which Med1 inhibits ferroptosis as well as inflammation have not been reported.

Our study identified a strong correlation between Nrf2 and Med1 expression, with significant Nrf2 upregulation in Lv-Med1 cells and downregulation in siMed1 cells. Additionally, the expression of HO-1, and NQO1, crucial downstream antioxidant enzymes of Nrf2 [36], displayed an expression pattern consistent with that of Nrf2. Moreover, the Nrf2 inhibitor, ML385, effectively counteracted the protective influence of Med1 against erastin-induced ferroptosis. These findings suggest that the inhibitory effects of Med1 on ferroptosis is mediated through the activation of Nrf2. Nrf2, a well-established inhibitor of ferroptosis [7, 37, 38], functions as a transcriptional regulator that enhances the transcription of numerous genes associated with antioxidant and cytoprotective properties by interacting with the antioxidant response element [39]. In this study, a notable augmentation in the expression of Nrf2 downstream antioxidant genes, namely HO-1, GCLC, and NQO1, was observed in Lv-Med1 cells following erastin exposure. Furthermore, elevated levels of HO-1 and NQO1 proteins were observed in mice overexpressing Med1. This finding further substantiates the activation of Nrf2 by Med1. Additionally, our data suggests that Med1 facilitates Nrf2 gene transcription, aligning with its established role as a transcriptional co-activator. Although there is evidence suggesting that Med1 may induce the activation of Nrf2 via the coactivator Ppargc1α [40, 41], additional research is required to ascertain the presence of direct interactions between Med1 and Nrf2.

In addition, the observed anti-inflammatory effect of Med1 in this study can also be attributed to Nrf2 upregulation. Primarily, upregulation of the antioxidant gene, HO-1, plays a significant role in anti-inflammatory functions [42]. Additionally, Nrf2 negatively regulates the NF-κB pathway through various mechanisms [43]. Furthermore, Nrf2 inhibits the transcriptional upregulation of proinflammatory genes, including IL-6 and IL-1β, induced by lipopolysaccharide [44]. Therefore, the anti-inflammatory effects of Med1 may similarly be mediated through activation of Nrf2.

The findings of our study suggest that Med1 has the ability to hinder ferroptosis and mitigate liver injury in cases of ALF through the activation of Nrf2. Nevertheless, the specific mechanism by which Med1 upregulates Nrf2 remains unclear. A limitation of this study is the lack of rigorous exploration aimed at elucidating this mechanism.

## Conclusions

In summary, our research findings indicate that Med1 effectively alleviates hepatic injury and improves the prognosis of LPS/GalN-induced ALF by inhibiting ferroptosis. This process is mediated by the activation of Nrf2, resulting in the upregulation of antioxidant genes and concurrent downregulation of proinflammatory cytokine transcription. In contrast to ferroptosis-specific inhibitors such as Fer-1, Med1 inhibited ferroptosis while attenuating inflammation. Strategies to target Med1 for ALF treatment show promise for research.

### Supplementary Information

**Additional file 1: Figure S1.** The animal experimental regimen applied in this study.

**Additional file 2: Figure S2.** The adenovirus vector used in the study.

**Additional file 3: Figure S3.** The mouse Med1 plasmid used in Ad-Med1 in the study.

**Additional file 4: Table S1.** Primer sequences for RT-qPCR.

## Data Availability

The datasets used and analyzed during the current study are available from the corresponding author upon reasonable request.

## Abbreviations

4‐HNE: 4-Hydroxynonenal
ALF: Acute liver failure
ACSL4: Acyl-CoA synthetase long chain family member 4
ALT: Alanine aminotransferase
AST: Aspartate aminotransferase
D-GalN: D-galactosamine
Fer-1: Ferrostatin-1
GCLC: Glutamate cysteine ligase catalytic
GSH: Glutathione
GPX4: Glutathione peroxidase 4
H&E: Hematoxylin & eosin
HO-1: Heme oxygenase-1
IL-6: Interleukin-6
LDH: Lactate dehydrogenase
Lv-Med1: Lentivirus overexpressing Med1
LPS: Lipopolysaccharide
MDA: Malondialdehyde
Med1: Mediator complex subunit 1
NQO1: NAD(P)H quinone oxidoreductase 1
Lv-NC: Negative control Lentivirus
Nrf2: Nuclear factor erythroid 2-related factor 2
Ppargc1α: Peroxisome proliferator-activated receptor gamma co-activator 1 alpha
PBS: Phosphate-buffered saline
PTGS2: Prostaglandin-endoperoxide synthase 2
ROS: Reactive oxygen species
siRNAs: Small interfering RNAs
SLC7A11: Solute carrier family 7, member 11
TNF-α: Tumor necrosis factor-α
TfR1: Transferrin receptor 1
