# Supplementary figures and images for: Med1 inhibits ferroptosis and alleviates liver injury in acute liver failure via Nrf2 activation
Source: Cell Biosci. 2024 Apr 27;14:54. doi: 10.1186/s13578-024-01234-4 (PMC11056072; doi:10.1186/s13578-024-01234-4)

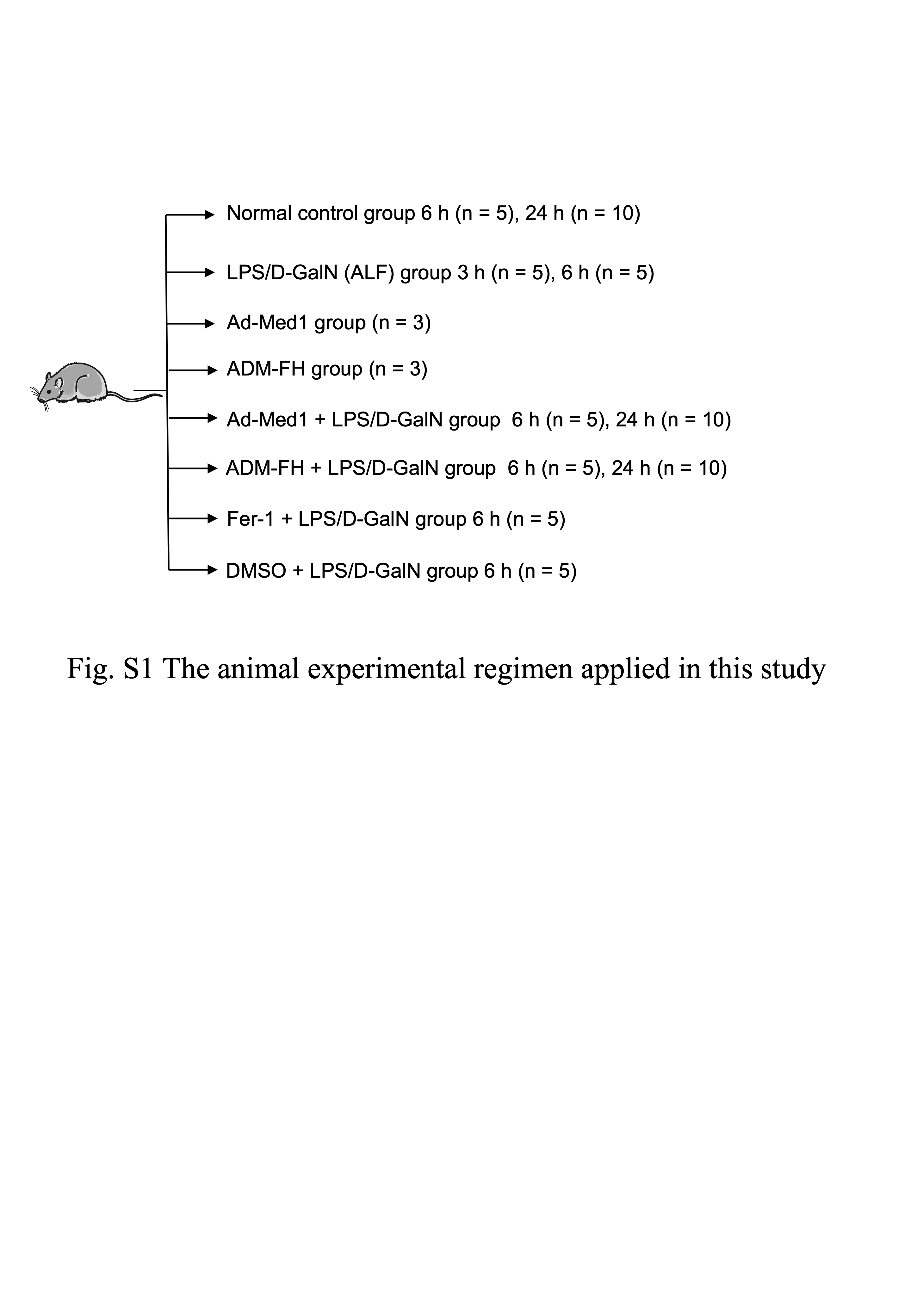

Supplement: Supplementary file 1 — Additional file 1: Figure S1. The animal experimental regimen applied in this study. [file 13578_2024_1234_MOESM1_ESM.tif]

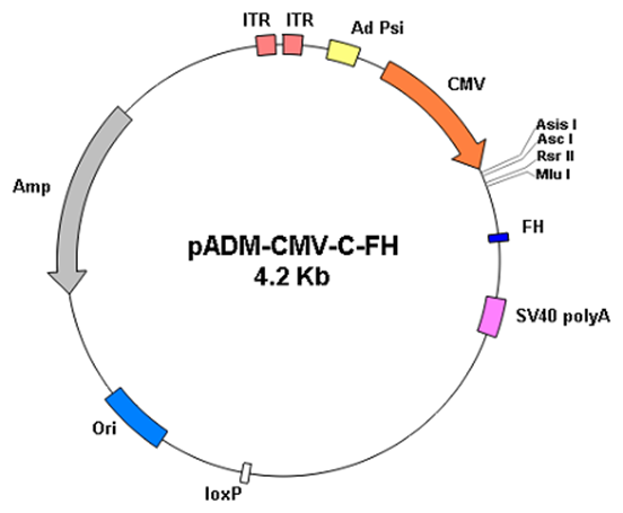

Supplement: Supplementary file 2 — Additional file 2: Figure S2. The adenovirus vector used in the study. [file 13578_2024_1234_MOESM2_ESM.tif]

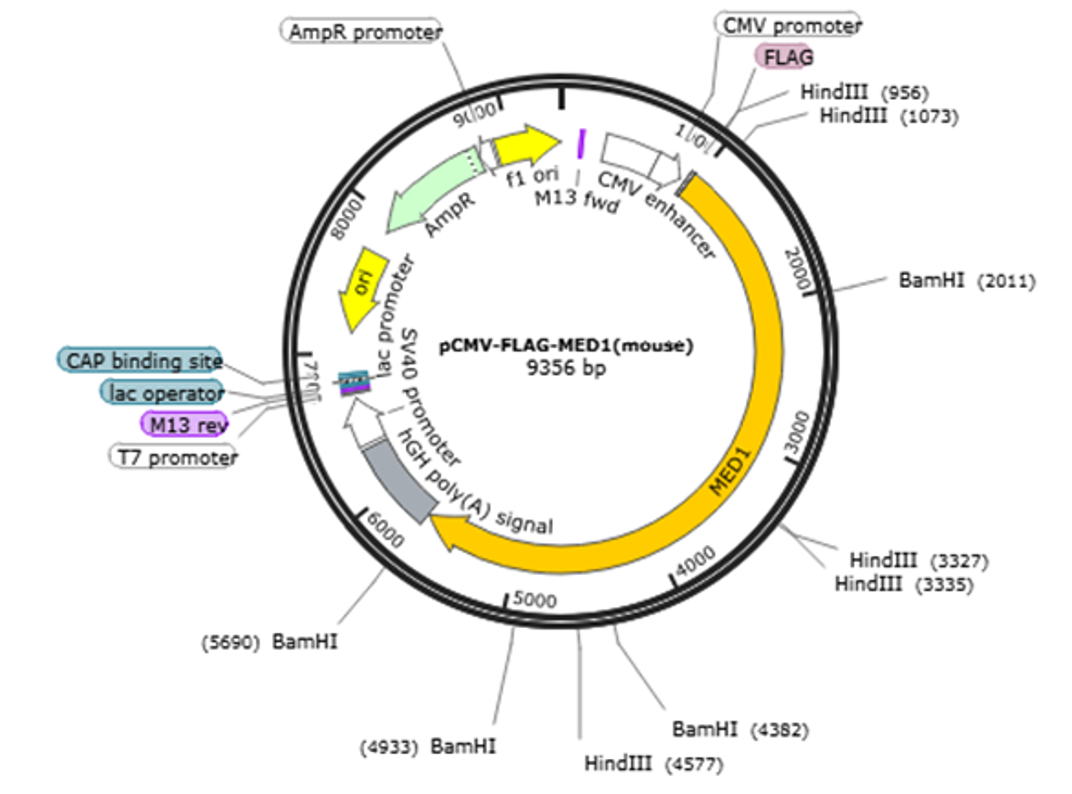

Supplement: Supplementary file 3 — Additional file 3: Figure S3. The mouse Med1 plasmid used in Ad-Med1 in the study. [file 13578_2024_1234_MOESM3_ESM.tif]
