# Supplementary material for: Med1 inhibits ferroptosis and alleviates liver injury in acute liver failure via Nrf2 activation
Source: Cell Biosci. 2024 Apr 27;14:54. doi: 10.1186/s13578-024-01234-4 (PMC11056072; doi:10.1186/s13578-024-01234-4)
Supplement: Supplementary file 4 — Additional file 4: Table S1. Primer sequences for RT-qPCR. [file 13578_2024_1234_MOESM4_ESM.docx]

Table S1. Primer sequences for RT-qPCR

| Gene name | | Forward sequences | Reverse sequences |
| --- | --- | --- | --- |
| Mouse | Med1 | GAGACTCCGCCCACTTACCTG | GGACACACTTCAAACTGGAGG |
|  | PTGS2 | CTGACCCCCAAGGCTCAAAT | TCCATCCTTGAAAAGGCGCA |
|  | SLC7A11 | GTCTGCCTGTGGAGTACTGT | ATTACGAGCAGTTCCACCCA |
|  | GPX4 | CGATCTGCATGCCCGATATG | GGCATCGTCCCCATTTACAC |
|  | TNF-α | CCTATGTCTCAGCCTCTTCT | CCTGGTATGAGATAGCAAAT |
|  | IL-6 | CCACTTCACAAGTCGGAGGCTT | CCAGCTTATCTGTTAGGAGA |
|  | Nrf2 | ATCTCCTAGTTCTCCGCTGC | GCTCATAGTCCTTCTGTCGC |
|  | Ppargc1α | GTGCCACCGCCAACCAAGAG | TTCCTCGTGTCCTCGGCTGAG |
|  | β-actin | CACTGTCGAGTCGCGTCCA | TGACCCATTCCCACCATCAC |
| Human | HO-1 | CCAGGCAGAGAATGCTGAGTTC | AAGACTGGGCTCTCCTTGTTGC |
|  | NQO1 | AGAAAGGATGGGAGGTGGTG | ATATCACAAGGTCTGCGGCT |
|  | GCLC | GGTGTGTTTCCTGGACTGATC | TGCGATAAACTCCCTCATCCA |
|  | β-actin | CGCGAGAAGATGACCCAGAT | CAGAGGCGTACAGGGATAGCA |
